# Food Security and Malnutrition Status in Patients with Cancer: An Australian Cross-Sectional Survey

**DOI:** 10.3390/nu18101599

**Published:** 2026-05-18

**Authors:** Kate Graham, Sandra Picken, Nicole Kiss, Rebecca Lindberg, Jenelle Loeliger, Belinda Steer

**Affiliations:** 1 Nutrition & Speech Pathology Department, Peter MacCallum Cancer Centre, Melbourne, VIC 3000, Australia; kate.graham@petermac.org (K.G.); jenelle.loeliger@petermac.org (J.L.); 2 Western & Central Melbourne Integrated Cancer Service, Melbourne, VIC 3000, Australia; sandra.picken@petermac.org; 3 Institute for Physical Activity and Nutrition, Deakin University, Geelong, VIC 3220, Australia; nicole.kiss@deakin.edu.au (N.K.); r.lindberg@deakin.edu.au (R.L.); 4 School of Exercise and Nutrition Sciences, Faculty of Health, Deakin University, Geelong, VIC 3220, Australia; 5 The Sir Peter MacCallum Cancer Department of Oncology, University of Melbourne, Parkville, VIC 3010, Australia

**Keywords:** food security, cancer, malnutrition

## Abstract

**Background**: Food insecurity is an important but under-recognised issue in cancer patients. It is linked to malnutrition and contributes to inequities in care. As there is minimal national population data available, this study aimed to assess the food security status of people receiving treatment for cancer in the state of Victoria, Australia. **Methods**: A multi-site point prevalence study was conducted in Victorian acute health services in July 2024. Adults receiving ambulatory treatment and multi-day stay inpatients were included. Patients were screened and assessed for malnutrition (using the Malnutrition Screening Tool and Global Leadership Initiative on Malnutrition criteria) and assessed for their food security status (using the Household Food Security Survey Module for Adults). **Results**: A total of 24 health services recruited 2121 adults with cancer. Overall, 6.9% experienced food insecurity, with the majority (52.4%) experiencing marginal food insecurity. No differences in food security status were observed between admitted and ambulatory patients, nor between metropolitan and regional/rural locations. Culturally and linguistically diverse (CALD) patients recorded higher rates of food insecurity compared to non-CALD patients (10.4% vs. 6.0%; *p* = 0.001). Patients who were food insecure had a higher prevalence of malnutrition compared to food secure patients (37.4% vs. 27.5%; *p* = 0.014). **Conclusions**: Although the prevalence of food security was low overall among patients with cancer, it was more pronounced in patients with malnutrition or from CALD backgrounds. To effectively address the issue of malnutrition in patients with cancer, food security must be considered as part of a multi-modal intervention.

## 1. Introduction

Food and nutrition security is vital for health at every life stage, supporting disease prevention and management. Over the last decade, a confluence of global forces—from the climate crisis to inflationary pressures—have exacerbated existing food and nutrition inequities [1]. Food insecurity, therefore, is a rising public health and clinical concern. The United States Department of Agriculture defines food insecurity as ‘the limited and uncertain availability of nutritionally adequate and safe foods, or limited or uncertain ability to acquire acceptable foods in socially acceptable ways’ [2]. The reported prevalence of moderate or severe food insecurity was 28.9% of the worldwide population in 2023, with higher rates in low- and middle-income countries, as well as in areas of conflict and disaster [1]. Food insecurity can increase risks for chronic diseases, including diabetes, heart disease, mental health conditions and cancer [3,4,5,6]. Moreover, food insecurity is associated with higher rates of all-cause and cause-specific mortality [7].

In high-income countries, despite advanced economic and health systems and high standards of living, food insecurity also occurs and contributes to health inequities. In Australia, according to 2023 data, it affects an estimated 13.2% of households [8]. Unlike other high-income countries, national surveillance of household food security does not form part of regular health or welfare monitoring in Australia. Community reports and cross-sectional studies in specific areas and population groups highlight some communities particularly at risk. For example, people from culturally and linguistically diverse (CALD) backgrounds, those living in remote areas, First Nations persons, and older people are all vulnerable groups at increased risk of food insecurity [8,9,10]. The impact of food insecurity on illness and disease in Australia is largely unknown.

A cancer diagnosis is accompanied by emotional, financial, and physical burdens, all of which can impact a person’s food security status [11,12]. Access to, and consumption of, adequate and nutritious food is essential for patients with cancer. Without this, malnutrition is likely to occur or be exacerbated if already present. Cancer-related malnutrition is a common condition occurring in up to 40% of people with cancer [13]. Its presence can have significant effects, including an increased risk of complications post-surgery, exacerbated treatment-related toxicities, poorer response to anti-cancer treatments, worse quality of life, and increased risk of mortality [14,15,16]. Therefore, addressing risk factors for malnutrition, including food insecurity, should be a priority for health professionals and health services treating people with cancer.

Estimates from other countries consistently report a higher prevalence of food insecurity in patients with cancer than the general population [12,17,18]. For example, in the United States, food insecurity is estimated to affect from 17 to 56% of people living with cancer [12,17,18,19,20] substantially driven by the financial burden of managing cancer and high rates of bankruptcy post-cancer diagnosis [21,22]. However, the prevalence of food insecurity in oncology populations in Australia is largely unknown. Given the relationship between food insecurity and malnutrition and inter-country differences in healthcare systems, understanding prevalence of food insecurity in Australian patients with cancer is important to improve patient outcomes. Therefore, this study aims to assess the food security status of people receiving treatment for cancer in the state of Victoria, Australia.

## 2. Materials and Methods

### 2.1. Study Design and Setting

This study reports data collected as part of a multi-centre observational malnutrition point prevalence study (PPS), following methodology previously reported [23,24]. A total of 24 health services across 35 locations in Victoria, Australia, from both public and private healthcare sectors were included. Recruitment and data collection occurred in July 2024. Sites collected data from multi-stay inpatients, as well as ambulatory chemotherapy and radiotherapy services. Site dietitians, or student dietitians who had reached clinical competency, completed data collection, including malnutrition screening and assessment and a food security questionnaire.

### 2.2. Participants and Eligibility

Eligible patients were aged ≥18 years, with a diagnosis of cancer, and were either admitted to hospital (minimum of two nights) for cancer treatment and/or related management, or were outpatients attending for radiotherapy, intravenous chemotherapy, or immunotherapy. All patients provided verbal consent to participate prior to data collection. Patients were excluded if they were admitted to intensive care units or emergency departments on the day of data collection, or were attending for day surgery, medical review, oral chemotherapy, or maintenance/hormonal therapy. Terminally ill patients with a life expectancy of less than one month were also excluded, as were patients who were unable to provide verbal consent. Multi-site ethics approval was received through Peter MacCallum Cancer Centre’s Ethics Committee (HREC/16/PMCC149).

### 2.3. Demographics and Clinical Data

Demographic data collected for each participant included sex at birth, age, living situation (alone, with family, or residential care), CALD background, and First Nations status. Clinical data collected included cancer diagnosis, presence of metastatic disease, current cancer treatment, treatment setting (inpatient or ambulatory), and treatment location (metropolitan or regional).

### 2.4. Food Security Assessment

All participants in the study were assessed for their food security status utilising the 10-question United States Department of Agriculture’s (USDA) Household Food Security Survey Module (HFSSM) for Adults [2]. The survey asks participants about their financial ability to purchase food, ability to purchase balanced meals, and if adults in the household were reducing portion sizes or skipping meals over the last 12 months. Affirmative answers scored one point each, with one or more affirmative answers identifying the participant as being food insecure. Scoring and categorisation of participants was conducted using the adapted Health Canada classification system [25,26]. Participants were categorised as being food secure if they reported no affirmative responses, including ‘prefer not to answer’. Marginal food security was defined as 1 affirmative answer, low food security by 2–4 affirmative answers, and very low food security by more than 5 affirmative answers. Studies in Canada and Australia have identified that a single positive response on the HHFSM indicates marginal food insecurity and as such this should be included as discrete category; therefore, this classification system was applied given its Australian setting of our study [25,26,27,28].

### 2.5. Malnutrition Screening, Assessment, and Diagnosis

All participants were screened using the Malnutrition Screening Tool (MST), which is validated for use in the oncology population [29,30,31]. Participants with an MST score of ≥2 were deemed at risk of malnutrition and underwent additional data collection to determine nutritional status. The Global Leadership Initiative on Malnutrition (GLIM) criteria for the diagnosis of malnutrition were applied as previously described [23,24,32]. In brief, the GLIM criteria require ≥ 1 phenotypic and ≥1 aetiological criteria to be present to diagnose malnutrition. The phenotypic criteria include low body mass index (BMI), unintentional weight loss, or reduced muscle mass. Height, weight, and weight history were collected from participants to calculate BMI and any weight loss over 6 months. Muscle status was assessed as described in the Patient-Generated Subjective Global Assessment (PG-SGA) [33]. Reduced muscle mass was met when a muscle deficit was present at ≥4 sites. The aetiological criteria include reduced food intake or inflammation. Food intake, including the degree of reduction compared to usual intake, was collected from each participant. Inflammation was determined by C-reactive protein (CRP) > 5 mg/L, a neutrophil-to-lymphocyte ratio (NLR) > 3.53, or presence of metastatic disease, which were all collected from medical records [23,24,32]. Blood tests had to have been collected within 7 days of data collection to be considered a valid measure.

### 2.6. Statistical Analysis

All data was entered electronically into a REDCap database at the time of collection. Once complete data was exported for analysis. All data were analysed using IBM SPSS Statistics(Version 27), Armok, NY, USA. Descriptive statistics are presented as the mean (SD). Analyses were undertaken using chi-square tests for equal proportions for categorical variables, or Fisher’s exact test where there were less than 5 cases in a cell. A two-sided *p*-value of <0.05 was considered statistically significant. In addition, binary logistic regression analyses were performed to assess the association between independent variables and the presence of food insecurity. Variables with a bivariate association, with the outcome of interest of *p* < 0.2, were entered into the model in a stepwise fashion. Variables were only retained in the model if they retained statistical significance.

## 3. Results

### 3.1. Participant Characteristics

A total of 2173 oncology patients consented to participate in the study. Collected data was cleaned to remove entries with missing or incomplete data (*n* = 52), resulting in 2121 participants included in the analysis. Of these, 341 (16.1%) were inpatients, with the remainder being treated in the ambulatory setting (*n* = 1780, 83.9%). The participant characteristics are shown in Table 1. The median age of participants was 67 years, the majority (78.0%) lived with family or carers, and were metro dwelling (78.3%).

### 3.2. Prevalence of Food Insecurity in Victorian Oncology Patients

**Figure 1:**
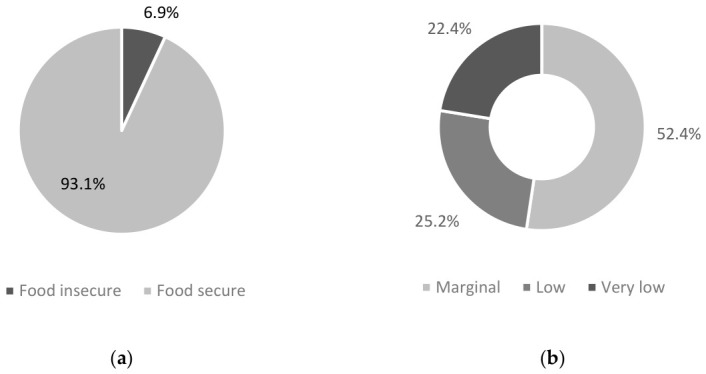
Prevalence of food insecurity in Victorian oncology patients. (**a**) Total food insecurity measured in included participants; (**b**) categorised analysis of level of food security within the food insecure population identified in 1 (**a**). These categories are determined by the sum of the affirmative responses to the HFSSM and assigned as per the scoring guide.

The majority of participants were classified as food secure (93.1%, *n* = 1974), with 6.9% (*n* = 147) identified as being food insecure (Figure 1a). A small portion of patients (*n* = 22, 1.0%) elected not to answer the food security questions and were counted in the food secure group. Of those classified as food insecure, most were assessed as having marginal food security (52.3% [77 marginal food security/147 total food insecure]), 25.1% had low food security (37/147), and 22.4% reported the most severe end of the continuum as very low food security (Figure 1b).

### 3.3. Food Insecurity by Participant Demographic

**Figure 2:**
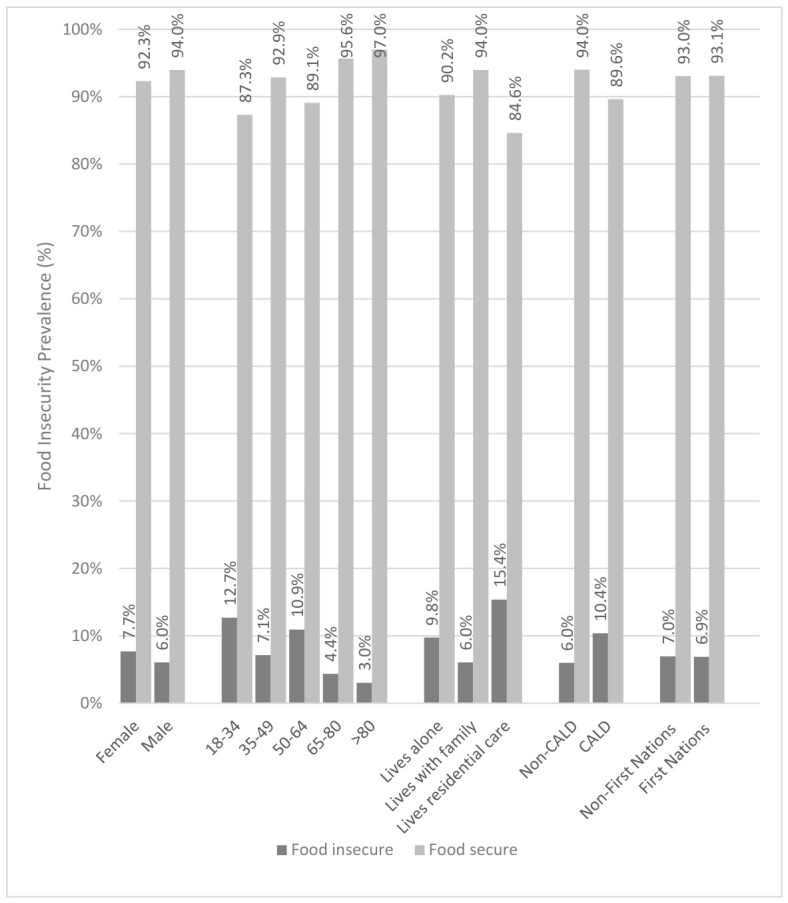
Food insecurity in Victorian oncology patients by demographic category. Total food insecurity was recorded against each participants demographic variable collected in the PPS.

Food insecurity according to participant demographic groups is shown in Figure 2. There were no significant differences in the level of food insecurity by sex (male = 6.0% vs. female 7.7%, *p* = 0.161). There was an overall significant difference in food insecurity by age group (*p* ≤ 0.001), with higher levels among participants aged 50–64 years (10.9%) and 18–34 years (12.7%). Participants who lived alone had significantly higher food insecurity compared to those living with family or carers (9.7% vs. 6.0%, *p* = 0.006). Similarly, participants from a CALD background experienced significantly higher levels of food insecurity compared to non-CALD participants (10.4% vs. 6.0%, *p* = 0.001). First Nations participants had similar levels of food insecurity (6.9%) to non-First Nation participants (7.0%, *p* = 1.0), although the overall number of First Nations participants was very small (*n* = 29, 1.4% of total participants).

In the multivariable logistic regression model, younger age was independently associated with higher odds of food insecurity (OR = 0.97 per year increase, 95% CI 0.96–0.98, *p* < 0.001), while living alone (OR = 1.99, 95% CI 1.35–2.92) and identifying as being from a CALD background (OR = 1.72, 95% CI 1.19–2.49) were each associated with increased odds of food insecurity. The model demonstrated acceptable fit (Hosmer–Lemeshow *p* = 0.435), although explained variance was modest (Nagelkerke R^2^ = 0.056), which is consistent with the multifactorial nature of food insecurity.

### 3.4. Food Insecurity by Cancer Care Setting

**Figure 3:**
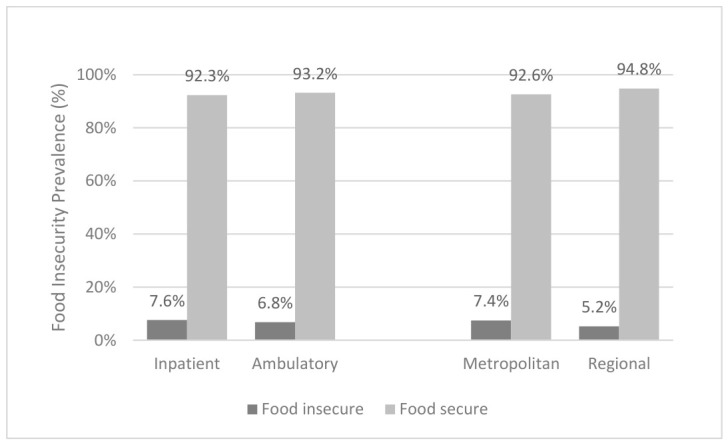
Food insecurity in Victorian oncology patients by cancer care setting. Total food insecurity was recorded for each participants location of cancer care treatment on the day of data collection—inpatient or ambulatory (radiotherapy, chemotherapy, immunotherapy)—or location of health service where care was delivered (metropolitan and regional).

Food insecurity was not significantly different in inpatients compared to ambulatory patients (7.6% vs. 6.8%, *p* = 0.664; (Figure 3)). Similarly, there were no significant differences in food insecurity between patients treated in regional/rural locations compared to those treated in metropolitan healthcare services (5.2% vs. 7.4%, *p* = 0.129).

### 3.5. Food Insecurity by Cancer Diagnosis

**Figure 4:**
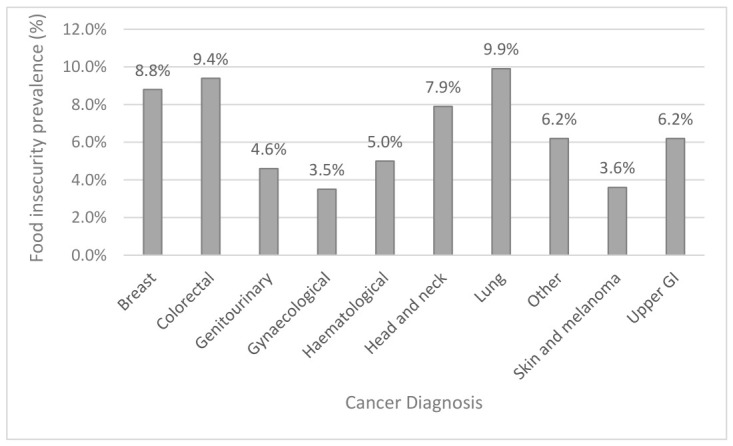
Food insecurity by cancer diagnosis in Victorian oncology population. Food insecurity expressed as a percentage of population for each cancer diagnosis. Upper GI diagnoses include endocrine and thyroid cancers; other diagnoses include bone and soft tissue, central nervous system, thoracic and abdominal, and secondary–primary unknown cancers.

The prevalence of food insecurity by cancer diagnoses is shown in Figure 4. Food insecurity was highest in participants with lung cancer (9.9%), colorectal cancer (9.4%), and breast cancer (8.8%), and lowest in participants with gynaecological cancer (3.5%); however, these differences were not statistically significant (*p* = 0.057).

### 3.6. Food Insecurity by Treatment Modality

**Figure 5:**
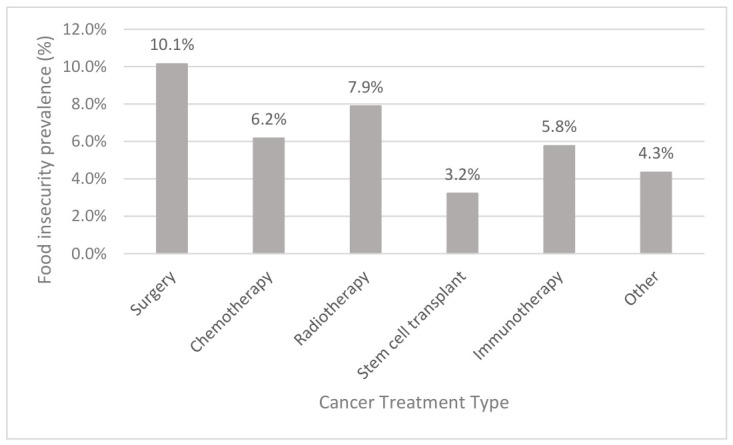
Food insecurity by cancer treatment in Victorian oncology population. Food insecurity expressed as a percentage of population for each cancer treatment. Chemotherapy refers to agents delivered intravenously; other refers to participants enrolled in clinical trials or receiving inpatient supportive care.

The prevalence of food insecurity by cancer treatment modality is shown in Figure 5.

Among these, participants who had surgery, a total of 10.1% (*n* = 30 of 287 total), were food insecure compared to those who did not have surgery (*n* = 117 of 1834). This was statistically significant (*p* = 0.012). Food insecurity was present in participants treated by other treatment modalities, such as chemotherapy (6.2%), radiotherapy (7.9%), stem cell transplant (3.2%), immunotherapy (5.8%), and other treatments (4.3%), but these were not statistically significant.

### 3.7. Impact of Food Insecurity on Malnutrition Prevalence

**Figure 6:**
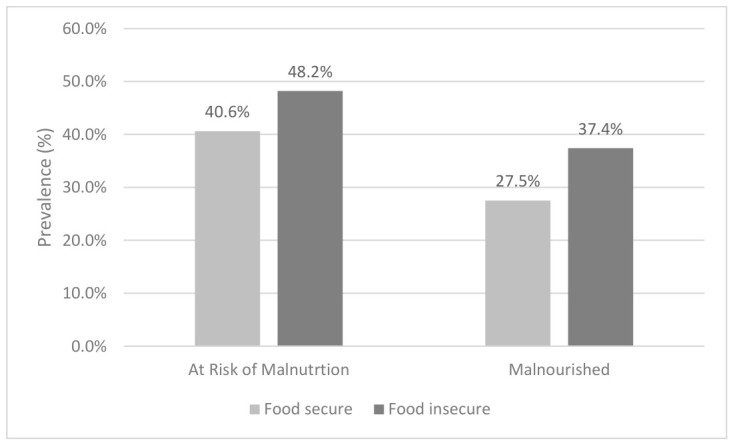
Malnutrition risk and malnutrition prevalence by food security classification: percentage of participants at risk of malnutrition or diagnosed with malnutrition by GLIM criteria in each food secure and food insecure groups.

The impact of food insecurity on malnutrition risk and GLIM-defined malnutrition prevalence is shown in Figure 6. Malnutrition risk was higher in food insecure participants (48.2%) compared to food secure participants (40.6%), but this was not statistically significant (*p* = 0.082). In contrast, GLIM-defined malnutrition was significantly higher in food insecure participants (37.4%) compared to food secure participants (27.5%, *p* = 0.014). The multivariate logistic regression model also identified that the presence of malnutrition was associated with increased odds of food insecurity (OR = 1.70, 95% CI 1.19–2.43). The model demonstrated acceptable fit (Hosmer–Lemeshow *p* = 0.435), although explained variance was modest (Nagelkerke R^2^ = 0.056), which is consistent with the multifactorial nature of food insecurity.

## 4. Discussion

To our knowledge, this is the first study to measure food insecurity prevalence in a large population of oncology patients undergoing active treatment in Australia. Further, it is one of the few studies worldwide conducted on household food security in patients affected by cancer outside of the US. This study found that almost seven percent of Victorian oncology patients were food insecure, with the majority (52.4%) assessed as having marginal food security. Food insecurity rates were significantly higher in those from a CALD background and those who lived alone. Food insecure participants also experienced a higher prevalence of malnutrition.

Placing our findings in context, we find that food insecurity prevalence in our study is considerably lower, at 7%, than rates published in other oncology populations, as well as in the Australian general population. Food insecurity prevalence in the general population is known to vary based on several factors, with higher rates commonly seen in racial and ethnic minority groups, younger female populations, those with lower incomes, and those living in rural/regional settings [34,35]. Our study included a heterogenous population of cancer patients; however, the majority were >50 years old living with family or carers in metropolitan locations, with only 22% identifying as being from a CALD background. Therefore, the relatively smaller numbers of known vulnerable groups may explain the lower prevalence of food insecurity among our participants compared to other studies. When looking at food insecurity prevalence by demographic groups, we did find statistically significant differences in comparisons between CALD participants and non-CALD participants (*p* = 0.001), and between those who lived alone and those who lived with family/carers (*p* = 0.006), as well as those of a younger age. This is in line with the current literature [8,9,10]. We did not find any difference in the prevalence of food insecurity in the First Nations population of our sample. This may be due to the very small numbers (*n* = 29) of First Nations participants recruited in the study; however it should be noted the percentage of total participants was 1.3% of the total study participants, which reflects the percentage of First Nations people in the Victorian population (1% in 2021 census) and suggests recruitment of First Nations people occurred at an appropriate rate [36]. Other states in Australia have higher proportions of First Nations people; for example, Queensland has 5.2% First Nations people in their population, so it would be of interest to assess food insecurity in the oncology population there [37]. It is also possible food insecurity prevalence was underreported in our First Nations group due to stigma and sensitivities, underscoring the need for repeated measures over time to ensure surveys are administered in a culturally appropriate manner.

Examining other reports in oncology populations, the prevalence in our cohort is most similar to a Canadian study, which included over 500,000 adults and reported a food insecurity prevalence of 10.2% [7]. In contrast, Charkhchi et al. (2018) studied a mixed population of US cancer patients, reporting a food insecurity prevalence of 22.6%, and Gany et al. (2014), who studied an underserved, low-income, predominantly immigrant sample of oncology patients, observed a food insecurity prevalence of 56% [19,34]. An Iranian study reported only 22.1% of the 240 cancer patients included were food secure, and found that economic status was significantly related to food insecurity [17]. The sociodemographic factors known to influence food insecurity in general also impact people with cancer; therefore, our reasons outlined above are also relevant to the differences between our study and other cancer populations. In addition, large-scale differences in healthcare systems in Australia compared to the US could account for the lower food insecurity prevalence in this Australian cancer population.

Data on food insecurity prevalence by cancer diagnosis or treatment modality may help prioritise resources and care. Our results suggest that there was no single cancer diagnosis with significantly higher food security, although we observed the highest prevalence in those with lung (9.9%), colorectal (9.4%), and breast cancer (8.8%). Berger et al. (2021) reported a similar prevalence of food insecurity in colorectal cancer patients in the US (10.1%), but a substantially higher prevalence in the head and neck cancer population (17.7%) than what we observed [38]. In a small cohort (*n* = 61) of Egyptian breast cancer patients, 47.5% were reported to be food insecure, suggesting this population may be at higher risk. However, high proportions of this population were unemployed at the time of the study and reported a household income below the poverty line, influencing this result [39]. We detected a higher prevalence of food insecurity in patients undergoing surgical treatments for their cancer. Food insecurity is associated with adverse operative outcomes and long-term survival for a range of cancer diagnoses, including upper gastrointestinal and breast cancers [40,41,42]. It is therefore important that further studies exploring the difference in food security by cancer diagnoses, treatment type, and outcomes are conducted.

The implications of an oncology patient being food insecure are not dissimilar to those of being malnourished, including a negative effect on recovery and compromised health-related quality of life [43,44]. When both food insecurity and malnutrition are present, it is likely that these adverse outcomes are compounded, however there is minimal literature describing this. Studies in the US have found the dietary intake of food insecure cancer patients may be nutritionally inadequate, with increased consumption of lower cost, lower quality foods and a lack of variety and essential nutrients [35]. Our results suggest that food insecurity often co-exists alongside the risk of malnutrition and is significantly associated with malnutrition prevalence, however the causal relationship requires further investigation. Whilst the co-existence of these two conditions is not surprising, data confirming this is an important step towards a better understanding of the interrelationship. This will help to inform future studies to investigate whether improving food security leads to meaningful improvements in nutritional status in cancer patients.

Screening for malnutrition is included in evidence-based guidelines and accreditation requirements for Australian hospitals and is consistently part of usual care [13,45,46,47]. However, screening for food insecurity is not part of routine practice. Our results suggest that in Australian oncology patients, screening for food insecurity should be considered, especially for those with identified malnutrition, from a CALD background, and/or those who live alone, regardless of their diagnosis or place of residence. Screening could be embedded in referral or admission processes, as well as included into nutrition assessments conducted by a dietitian. Our study has used the full 10-question survey utilised primarily in research studies. This would be burdensome for clinicians and participants to complete among the many other questions required during admissions or treatment. Instead, the two-item screening tool, the Hunger Vital Sign (HVS), could be utilised [48]. The HVS has been validated in large adult and paediatric cohorts and shown to have a high degree of sensitivity and specificity [49,50]. Its validity in the oncology population is not as well studied, despite recommendations to use HVS to reduce respondent burden [48]. As such, further studies comparing HVS to HHFSM in the Australian oncology population to address this gap should be conducted. Completion of screening for food insecurity would indicate which patients may benefit from information on where to locally access support to help mitigate food insecurity and crisis relief, as well as referral to oncology social workers. Unlike the US, Australia has no large-scale food stamps, school meals, and/or common referral pathways if food insecurity is recognised in healthcare settings [51]. However, considering the stigma and suboptimal nutritional profile of the foods commonly available in food relief settings, food security interventions that meet the needs of cancer patients merit attention [10,52]. This study, combined with other existing Australian data, could be used to inform health policy to address this significant issue, especially in the cancer setting. Inclusion of screening for food insecurity into healthcare settings would help to identify the issue; however, culturally appropriate, accessible food relief programmes across the country are required to address the issue. Due to considerable differences in demographics across Australia, further state-based or national studies would be required to better understand the vulnerable populations and inform future health policy to address this issue.

The large numbers included in our sample, along with the heterogenous population, are strengths of our study; however, there are limitations. Firstly, the lack of socioeconomic data, including household income, employment disruption, and cancer-related financial toxicity, is a limitation of our study and may act as a confounding factor to the findings. While our data has supported the existing literature regarding some of the known high-risk groups, it has not been able to demonstrate the link between low income and food insecurity in an Australian oncology population; future studies must include this data to ensure the findings can be used to inform health policy and lead to positive change. Additionally, due to the nature of data collection occurring in shared hospital rooms or large, open clinic spaces, it is possible that social desirability bias, stigma, or sensitivity to the questions may have influenced participants’ answers, resulting in an underestimation of food security prevalence and/or the severity of this in our study. Underreporting in food insecurity questionnaires that use direct questioning, such as the HHFSM, is an established drawback [53]. Future studies should ensure privacy for participants by considering the use of self-administered questionnaires or indirect questioning methodology, e.g., list experiments to reduce potential biases and underreporting [53]. Finally, the data were collected as part of a point prevalence study, and it is not possible to identify at what point a patient was in their cancer treatment, or if food security status changes throughout treatment. This limits the ability to determine if there are certain timepoints where food insecurity prevalence is highest or if a cancer diagnosis worsens a patient’s food security status. Future studies should assess food security from diagnosis through to survivorship to determine the effect of a cancer diagnosis, including financial toxicity, on an individual’s food security.

## 5. Conclusions

In conclusion, this is the first reported measure of food insecurity in an Australian oncology population. Whilst the prevalence of food insecurity was lower than anticipated, it remains an important first step to understanding this issue for oncology patients and demonstrates the need for further consideration of food security screening in routine care. Further studies are required for a deeper understanding of the drivers of this issue in oncology patients and could include collection of income status alongside food security to further understand and explain this significant issue. Ultimately, screening alone is not enough and the development and availability of acceptable interventions to address food insecurity are required for positive outcomes in oncology patients to be realised.

## Figures and Tables

**Table 1:** Participant characteristics (*n* = 2121).

Category	*n* (%)
Sex at birth	
Male	991 (46.7%)
Female	1130 (53.3%)
Age	
18–34 years	63 (3.0%)
35–49 years	266 (12.6%)
50–64 years	677 (31.9%)
65–80 years	917 (43.2%)
>80 years	198 (9.3%)
Social Situation	
Lives with family/carer	1654 (78.0%)
Lives alone	441 (20.8%)
Lives in residential care	26 (1.2%)
Identifies from a CALD background *	
Yes	462 (21.8%)
No	1652 (77.9%)
Unknown	7 (0.3%)
First Nations status	
First Nations person	29 (1.4%)
Non-First Nations person	2082 (98.2%)
Unknown	10 (0.4%)
Location	
Metro	1662 (78.3%)
Regional	459 (21.7%)

* Supplementary Table S1 provides details on the CALD backgrounds of CALD identifying patients included in the study.

## Data Availability

The data is owned by the Victorian Department of Health. Data will be shared on request to the corresponding author with permission of the Victorian Department of Health.

## Supplementary Materials

The following supporting information can be downloaded at: https://www.mdpi.com/article/10.3390/nu18101599/s1, Table S1 Detailed outline of CALD backgrounds of CALD identifying patients included in the study.

## Abbreviations

The following abbreviations are used in this manuscript:

CALD	Culturally and Linguistically Diverse
PPS	Point Prevalence Study
USDA	United States Department of Agriculture
HFSSM	Household Food Security Survey Module
MST	Malnutrition Screening Tool
HVS	Hunger Vital Sign
GLIM	Global Leadership Initiative on Malnutrition
PG-SGA	Patient-Generated Subjective Global Assessment
CRP	C-Reactive Protein
NLR	Neutrophil-to-Lymphocyte Ratio
