# Supplementary material for: Food Security and Malnutrition Status in Patients with Cancer: An Australian Cross-Sectional Survey
Source: Nutrients. 2026 May 18;18(10):1599. doi: 10.3390/nu18101599 (PMC13209256; doi:10.3390/nu18101599)
Supplement: Supplementary file 1 [file nutrients-18-01599-s001.zip › nutrients-4285606-supplementary.pdf]

Supplementary Table S1: Detailed outline of CALD backgrounds of CALD identifying patients included in the study.

CALD background	Number of patients
Italian	44
Greek	39
Mandarin	33
Vietnamese	32
Indian (inc. Punjabi, Sikh)	23
Philippines	21
Croatian	18
Cantonese	13
Arabic	12
Macedonian	12
German	9
Turkish	9
Maltese	8
Spanish	9
Sri Lakan	8
Chinese	7
Polish	7
Serbian	7
Dutch	6
Malaysian	6
Russian	6
Indonesian	5
Scottish	5
Albanian	4
Chilean	4
Egyptian	4
French	4
Hungarian	4
Samoan	4
Bosnian	3
Cambodian	3
Ethiopian	3
East Timorese	3
Hindi	3
Lebanese	3
New Zealand	3
Pakistani	3
Persian	3
South African	3
Thai	3
Afghanistan	2
Argentina	2
Assyrian	2
Bangladesh	2
Colombian	2
Fijian	2

Japanese	2
Māori	2
Nepalese	2
Portugese	2
Romanian	2
Sinalese	2
Somalian	2
Sudanese	2
African	1
Assyrian Neo-Aramaic	1
Belgium	1
Bengali	1
Bulgarian	1
Burmese	1
Burundi	1
Catholic	1
Cook Islands	1
Creole	1
Cook Islands	1
Czech	1
Dari	1
Did not specify	1
Dutch & Portuguese	1
French/Creole	1
Georgian	1
Hazara	1
Hebrew	1
Hong Kong	1
Hungary & Poland	1
Iranian	1
Iraq	1
Korean & German	1
Laos	1
Mauritius	1
Mexican	1
Monastry	1
Myanmar	1
Netherlands	1
Pacific Islander	1
Slovenia	1
South Sudan	1
Spanish	1
Swedish	1
Telugu	1
Trigina & Hamarik	1
Ukrainian	1
Ukrainian & polish	1
Zambian	1
